# Effect of repetitive transcranial magnetic stimulation-assisted training on lower limb motor function in children with hemiplegic cerebral palsy

**DOI:** 10.1186/s12887-024-04605-5

**Published:** 2024-02-22

**Authors:** Yan He, Qi Zhang, Ting-Ting Ma, Yan-Hua Liang, Rong-Rong Guo, Xiao-Song Li, Qian-Jin Liu, Tian-Yang Feng

**Affiliations:** 1 Department of Paediatric Physiotherapy, China Rehabilitation Research Center, Beijing Bo’ai Hospital, No. 10 of Jiaomen North street, Fengtai District, Beijing, 100068 China; 2 Capital Medical University School of Rehabilitation Medicine, No. 10 of Jiaomen North Street, Fengtai District, Beijing, 100068 China

**Keywords:** Hemiplegic cerebral palsy, Lower limb, Motor function, Rehabilitation, Repetitive transcranial magnetic stimulation

## Abstract

**Objective:**

To explore the effect of repetitive transcranial magnetic stimulation (rTMS)-assisted training on lower limb motor function in children with hemiplegic cerebral palsy (HCP).

**Method:**

Thirty-one children with HCP who met the inclusion criteria were selected and randomly divided into a control group (*n* = 16) and an experimental group (*n* = 15). The control group received routine rehabilitation treatment for 30 min each time, twice a day, 5 days a week for 4 weeks. Based on the control group, the experimental group received rTMS for 20 min each time, once a day, 5 days a week for 4 weeks. The outcome measures included a 10-metre walk test (10MWT), a 6-minute walk distance (6MWD) test, D- and E-zone gross motor function measurements (GMFM), the symmetry ratio of the step length and stance time and the muscle tone of the triceps surae and the hamstrings (evaluated according to the modified Ashworth scale), which were obtained in both groups of children before and after treatment.

**Results:**

After training, the 10MWT (*P* < 0.05), 6MWD (*P* < 0.01), GMFM (*P* < 0.001) and the symmetry ratio of the step length and stance time of the two groups were significantly improved (*P* < 0.05), there was more of an improvement in the experimental group compared with the control group. There was no significant change in the muscle tone of the hamstrings between the two groups before and after treatment (*P* > 0.05). After treatment, the muscle tone of the triceps surae in the experimental group was significantly reduced (*P* < 0.05), but there was no significant change in the control group (*P* > 0.05).

**Conclusion:**

Repetitive TMS-assisted training can improve lower limb motor function in children with HCP.

## Introduction

Cerebral palsy (CP) is a group of conditions characterised by motor dysfunction due to non-progressive brain injury of the developing foetus or in infants [1]. Cerebral palsy is the most common cause of childhood-onset lifelong physical disability in most countries [2]. Presently, there are over 5 million patients with CP in China, with an annual increase of approximately 40,000–50,000 cases [3]. Generally, hemiplegic CP (HCP) accounts for 38% of all cases of CP [4]. These children’s motor and sensory disorders mainly occur on one side of the body [5] and are characterised by the asymmetry of spatiotemporal kinematics parameters, such as the step length and support period [6]. Consequently, the physical and mental health and the quality of life of children with CP are affected, imposing a heavy economic and mental burden on both society and families [7, 8].

Over 80% of all children with CP present brain structural abnormalities on magnetic resonance imaging, with periventricular white matter softening, deep grey matter damage and brain dysplasia being the most common [9]. The continuing presence of abnormalities observed on imaging seriously affects the neurological development and clinical symptom recovery of children. Abnormal posture and movement disorders are the core symptoms of CP. The symptoms of HCP in children are determined by the different areas of brain tissue damage, which manifest differently. Regular rehabilitation is sometimes combined with other therapies, including mental and speech training, physical therapy, acupuncture, massage, bracing and plaster orthopaedics [10–13]. Orthopaedic surgery is only indicated for those with spasticity who are mentally competent and for whom non-surgical treatment is not effective [14]. Currently, the clinical treatment methods for paediatric CP include medication, surgery, acupuncture, physical therapy and rehabilitation [4]. Theoretically, compared with conventional regulation and remodelling of the central nervous system (CNS) by improving the function of the peripheral organs from the bottom to the top, a treatment that acts directly on the cerebral cortex or nerve cells can promote the development of the nervous system and compensate for the original dysfunction [15].

Repetitive transcranial magnetic stimulation (rTMS) is a non-invasive brain stimulation technology [16] that has been applied to the monitoring, evaluation and treatment of nervous system disease and provides a new way to explore the structure and function of the brain [17, 18]. According to domestic and international research, the application guidelines recommended by the International Federation of Clinical Neurophysiology and expert consensus on TMS therapy for CP in China, rTMS is both safe and feasible [3, 19–21]. The technique has achieved significant results in the rehabilitation of adult patients with stroke [22, 23] and can significantly improve gait, balance and lower limb function in such patients [24]. In recent years, rTMS has gradually been applied to the rehabilitation treatment of children with CP. However, most studies have focused on the effect of rTMS on upper limb function [25–28]. Accordingly, the present study mainly observed the rehabilitation effect of rTMS-assisted training on lower limb motor function in children with HCP to guide clinical treatment.

## Materials and methods

### Sample and recruitment

#### Study and participants

**Fig. 1:**
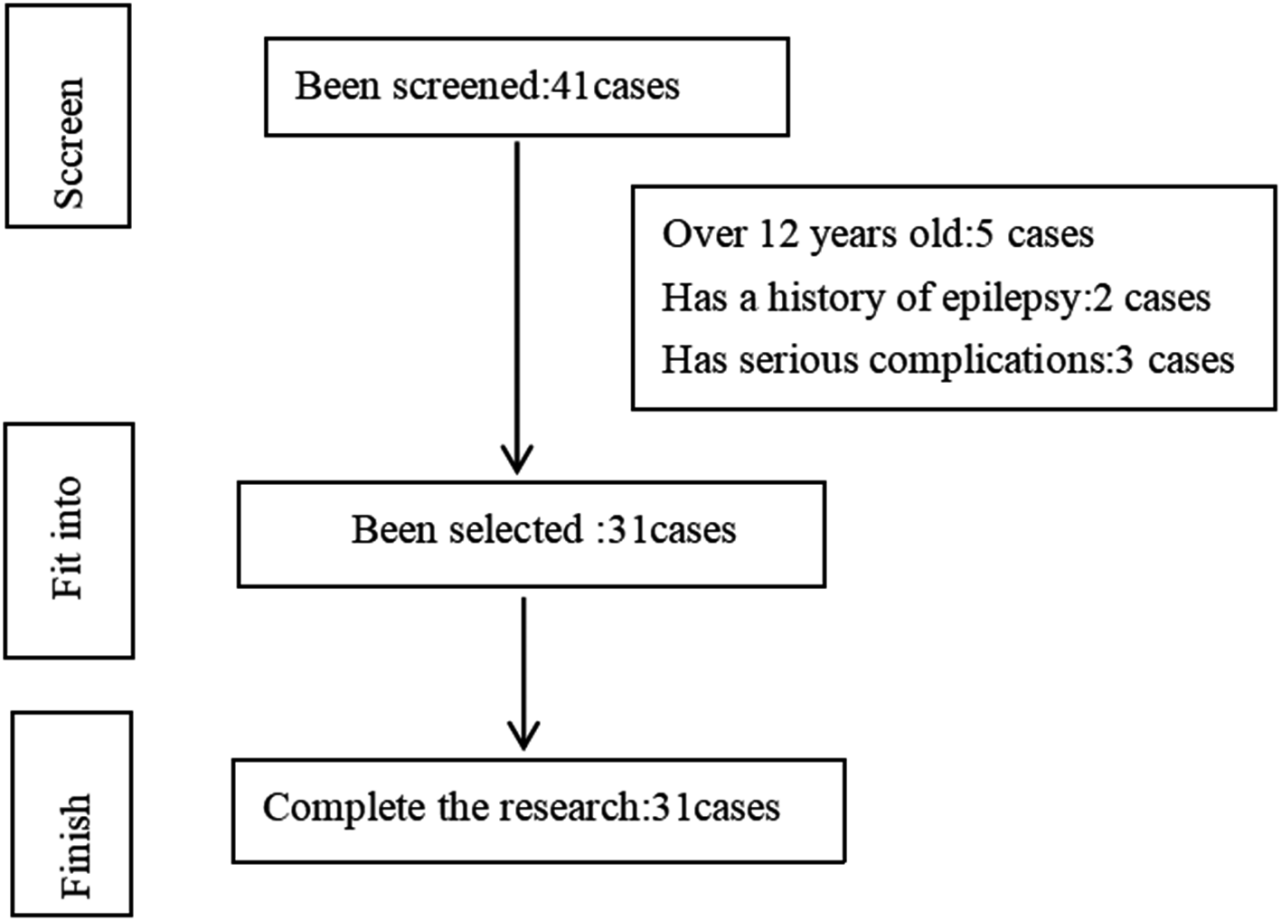
The inclusion process of participants

This study was a randomised controlled study. Thirty-one children with HCP who met the study’s inclusion criteria and were treated in the Department of Pediatric Physiotherapy of the China Rehabilitation Research Center, Beijing Boai Hospital between August 2021 and February 2023, and who met the diagnostic criteria for HCP in the *2015 Chinese Guidelines for the Rehabilitation of CP*, were selected. The participant inclusion process is shown in Fig. 1.

#### Eligibility criteria

Inclusion criteria: patients aged 3–12 years; Gross Motor Function Classification System (GMFCS) grade I–III [29]; ability to understand and obey the instructions of the therapist.

Exclusion criteria: patients with severe comorbidities (full or partial blindness, lower limb deformities); those with a history of epilepsy; those with metal implants in their bodies; those who received a botulinum toxin injection or surgery within 6 months before participating in the study; those who underwent surgeries related to lower limb motor function and muscle tone, such as selective posterior rhizotomy.

Elimination and dropout criteria: patients who automatically terminated treatment and those who were unable to continue treatment due to unexpected reasons (e.g. sudden deterioration in a condition, requirement for other treatments or death) during the research process.

This study followed the basic principles of the Declaration of Helsinki and was approved by the Ethics Committee of the China Rehabilitation Research Center (number, 2021-060-1). A sufficient explanation was given to each child and their parents about the feelings they would experience and possible side effects, and all the children’s families provided signed informed consent.

### Intervention

The children were divided into a control group and an experimental group by random number table grouping, with 16 cases in the control group and 15 in the experimental group. The control group was treated with conventional physical therapy, and the experimental group was treated with rTMS, based on the control group. The rehabilitation treatment plan was jointly formulated by the attending physician and rehabilitation therapist based on the patient’s condition. Conventional rehabilitation treatment was provided by rehabilitation therapists.

#### Conventional physical therapy

Conventional physical therapy included passive movement exercises, muscle contraction and extension, weight-bearing and centre-of-gravity transfer training on the affected lower limb, balance training, gait training and task-oriented activity training [30]. Passive activity and traction training were delivered by a therapist and an apprentice and mainly involved the muscles of the gastrocnemius, hamstring and adductor, to temporarily relieve muscle tension and maintain a normal joint range of motion. Strength enhancement training included muscle strength enhancement training for the tibialis anterior, gluteus medius and gluteus maximus; weight-bearing and centre-of-gravity transfer training for hemiplegic lower limbs; orthostatic balance training and gait correction training. Based on the different functional conditions of each child, the therapist provided assistance or verbal guidance, allowing the child to walk in front of a posture correction mirror and gradually control and correct their walking posture through visual feedback. Task-oriented activity training, such as walking up and down stairs, was also conducted. Each exercise was performed for 30 min, twice a day, 5 days a week for 4 weeks.

#### Repetitive transcranial magnetic stimulation therapy

The experimental group received rTMS via a transcranial magnetic stimulator (Henan Youde Medical Equipment Co., Limited Liability Company [Ltd.], China) and a navigator (Brain Science Tools BV, Netherlands) [3]. The coil of the transcranial magnetic stimulator was figure-of-eight-shaped, with a maximum magnetic field strength of 5.0 T. The operation steps were as follows. (1) The Navigator was matched and used for positioning, matching the brain of the child with the brain model of rTMS. (2) Determination of resting motor threshold (RMT): The child was placed in a comfortable sitting or lying position, and the stimulation coil was placed in the primary motor cortex on the non-affected side of the head (1–4 cm from the skull apex of the non-affected side). The single-pulse stimulation mode was used at 80% of the maximum output intensity; the coil was moved back and forth in this area to find the position of the maximum motor-evoked potential (MEP) of the tibialis anterior in the resting state. At least 5 out of 10 stimuli induced an amplitude of MEP > 50 µV in the tibialis anterior, and the minimum magnetic stimulation intensity was the RMT. This position was set as the target for TMS, and the Navigator automatically recognised and mapped the target to the cerebral cortex model. (3) Placement of the figure-of-eight-shaped coil. The figure-of-eight-shaped coil was fixed according to the Navigator at a 45° angle to the horizontal plane while keeping the distance between the stimulation point and the target within 20 mm (preferably within 10 mm) to ensure the accuracy and effectiveness of TMS. (4) Stimulation parameters: Using a repetitive stimulation mode, the stimulation intensity was set to 90% RMT, with a stimulation frequency of 1 Hz. After continuous stimulation for 20 s, the stimulation was repeated at an interval of 20 s. Each stimulation treatment was performed for 20 min, once a day, 5 days a week for a total of 4 weeks.

### Outcome measures

All measures were assessed by two therapists who could not distinguish between the subgroups to ensure the accuracy of the study.

#### Ten-metre walk test (self-selected speed)

A 10-metre walk test (10MWT) was used to evaluate walking speed. The therapist instructed the participants to complete three tests on a 14-metre walkway at an appropriate and comfortable speed. To avoid the impact of acceleration and deceleration, only the walking time of the middle 10 m was recorded, and the average was taken to calculate the participants’ self-selected walking speed.

#### Six-minute walk distance

A 6-minute walk distance (6MWD) test was used to evaluate walking endurance. The participants were instructed to walk and turn at an appropriate speed on a 10-metre walkway for 6 min, and the distance travelled was recorded. The participants could wear or use any ankle and foot orthotics or walking aids they required.

#### Gross motor function measurement

The Cronbach’s α of the GMFM was 0.96 [31], which showed good reliability. The D-zone (standing) and E-zone (walking, running and jumping) of the GMFM were used for the GMFM, with a total score of 111 points for a total of 37 items. The higher the score, the better the motor function.

#### Symmetry ratio of gait spatiotemporal parameters

Data on the step length and stance time of both lower limbs were obtained using a flat foot pressure assessment system (Tongtong [Beijing] Technology Co., Ltd.). The symmetry ratio was calculated according to the formula ‘Symmetry ratio = V affected side/V healthy side’ [32], where V represents the spatiotemporal parameters of each gait. When the ratio is 1, it indicates that both lower limbs are completely symmetrical. The further the deviation from 1, the worse the symmetry. This study mainly observed the symmetry ratio of lower limb step length and standing time before and after treatment in the two groups of participants.

#### Muscle tone of the triceps surae and the hamstrings

The modified Ashworth scale was used to evaluate the muscle tone of the triceps surae and the hamstrings on the hemiplegic side. There were 4 (IV) levels in total, with 0 being normal. The higher the level, the higher the muscle tone of the child.

### Statistical analysis

Data processing was performed using the SPSS 26.0 statistical software package. The Shapiro–Wilk test was used to test for normality. A paired *t*-test was used for intra-group comparisons, and an independent samples *t*-test was used to compare the measurement data in the two groups. Fisher’s exact test was used to compare the enumeration data. A value of *P* < 0.05 indicated a significant difference.

## Results

All 31 participants completed this study, and no serious adverse events were reported. The specific results are as follows.

### General characteristics

The children were divided into a control group and an experimental group by random number table grouping, with 16 cases in the control group and 15 in the experimental group. There were no significant differences in age, gender, hemiplegic side and GMFCS grade between the two groups (*P* > 0.05) (Table 1).

**Table 1:** Comparison of general characteristics of the subjects between two groups

Demographic data	Control group (*n* = 16)	Experimental group (*n* = 15)	t/x2-value	*P*-value
Age (years)	7.38 ± 2.92	6.47 ± 2.42	0.940	0.355
Gender (male/female, n)^*^	11/5	12/3		0.685
Hemiplegic side (left/right, n) ^*^	9/7	10/5		0.716
GMFCS (I/II/ III) ^*^	6/7/3	5/9/1		0.621

* “Gender”, “Hemiplegic side” and “GMFCS” are calculated using Fisher’s exact probability method, no chi-squared values

### Walking ability

There was no significant difference in the 10MWT and 6MWD scores between the two groups before treatment (*P* > 0.05). After treatment, the 10MWT and 6MWD results in both groups significantly increased (*P* < 0.01), and the value in the experimental group was significantly higher than in the control group (*P* < 0.05). The results are shown in Table 2.

**Table 2:** Comparison of 10MWT and 6MWD between the two groups of participants at different times

Group	N	Before treatment	After treatment	t-value	p-value
10MWT-C (m/s)	16	0.40 ± 0.09	0.58 ± 0.10	-15.164	< 0.001
10MWT-E (m/s)	15	0.38 ± 0.09	0.67 ± 0.14	-17.020	< 0.001
t-value		-0.674^*^	-2.208		
p-value		0.505	0.035		
6MWD-C (m)	16	91.83 ± 12.88	133.28 ± 19.00	-14.492	< 0.001
6MWD-E (m)	15	84.67 ± 8.90	199.81 ± 37.35	-15.872	< 0.001
t-value		-0.681^*^	2.744		
p-value		0.502	0.010		

*Note* “-C” means control group, “-E” means Experimental group. “*” denotes unequal variance, using continuous correction t-test

### Gross motor function measurement

When comparing the two groups, there was no significant difference in the GMFM scores before treatment (*P* > 0.05). After treatment, there was a significant increase in scores between the two groups (*P* < 0.01), with the score of the experimental group being significantly higher than that of the control group (*P* < 0.01). The results are shown in Table 3.

**Table 3:** Comparison of GMFM between the two groups of participants at different times

Group	N	Before treatment	After treatment	t-value	p-value
GMFM-C	16	76.69 ± 7.09	87.13 ± 5.76	-10.757	< 0.001
GMFM-E	15	78.44 ± 6.95	96.75 ± 7.01	-69.502	< 0.001
t-value		0.667^*^	4.049		
p-value		0.510	< 0.001		

*Note* “-C” means control group, “-E” means Experimental group. “*” denotes unequal variance, using continuous correction t-test

### Symmetry ratio of the step length and stance time

There was no significant difference in the symmetry ratio of the step length and stance time between the two groups before treatment (*P* > 0.05). However, after treatment, the symmetry ratio of the step length and stance time of the two groups were significantly improved (*P* < 0.05), and the score in the experimental group was significantly better than in the control group (*P* < 0.05). The results are shown in Table 4.

**Table 4:** Comparison of symmetry ratio of step length and stance time between the two groups of participants at different times

Group	N	Before treatment	After treatment	t-value	p-value
SRSL-C	16	0.78 ± 0.09	0.87 ± 0.09	-3.017	0.009
SRSL-E	15	0.76 ± 0.16	0.93 ± 0.05	-4.231	0.001
t-value		-0.383	2.321		
p-value		0.705	0.028		
SRST-C	16	1.07 ± 0.04	1.04 ± 0.02	2.419	0.029
SRST-E	15	1.09 ± 0.07	1.02 ± 0.03	4.514	< 0.001
t-value		1.197	-2.445		
p-value		0.241	0.021		

*Note* “SRSL” means symmetry ratio of step length, “SRST” means symmetry ratio of stance time. “-C” means control group, “-E” means Experimental group

### Muscle tone of the triceps surae and hamstrings

When comparing the two groups, there was no significant difference in the scale of muscle tone of the triceps surae and hamstrings before treatment (*P* > 0.05). After treatment, the scale of muscle tone of the triceps surae in the experimental group was significantly reduced (*P* < 0.01), but there was no significant change in the control group (*P* > 0.05). The scale of muscle tone of the triceps surae in the experimental group was better than in the control group (*P* < 0.05). There was no significant change in hamstring muscles between the two groups (*P* > 0.05). The results are shown in Table 5.

**Table 5:** Comparison of muscle tone of triceps surae and hamstrings between the two groups of participants at different times

Group	N	Before treatment			After treatment			p-value
		I	I^+^	II	I	I^+^	II	
MTTS-C	16	5	9	2	6	8	2	1.00
MTTS-E	15	4	10	1	12	3	0	0.009
p-value			1.00			0.038		
MTH-C	16	5	10	1	6	9	1	1.00
MTH-E	15	3	11	1	5	9	1	0.833
p-value			0.836			1.00		

*Note* “MTTS” means Muscle tone of triceps surae, “MTH” means Muscle tone of hamstrings. “-C” means control group, “-E” means Experimental group

## Discussion

In this study, we observed the effect of rTMS combined with conventional physical therapy on the lower limb motor function of children with HCP. The results showed that the application of rTMS had a significant therapeutic effect on the lower limb motor function of children with HCP and was well tolerated. This provides support for the application of rTMS in children with HCP.

The plasticity of the brain is the theoretical basis of physical therapy for children with HCP, who are mainly characterised by developmental disorders in posture and movement. Spasticity, posture and motor control disorders greatly affect the daily lives and learning of children with HCP. Therefore, early active and effective rehabilitation treatment is of great importance. At present, physical therapy for children with HCP mainly focuses on various manipulations and instruments, lacking direct stimulation to the functional areas of the cerebral cortex. Repetitive TMS compensates for the deficiency of conventional physical therapy, as it can inhibit the non-affected side or stimulate the affected side to promote the recovery of balance, rebuild hemispheric balance and improve lower limb motor function in children with HCP [33]. Repetitive TMS can be used in the representative areas of the motor and non-motor cortexes. Through repeated, continuous and regular stimulation of the brain, it can exert a cumulative effect and produce local and remote effects on brain activity to achieve the regulation function of cortical excitability, help reconstruct the functional areas of the cortex and regulate neuroplasticity of the cerebral cortex [33]. Due to its painless, non-invasive and safe characteristics, it has been widely studied in neurological rehabilitation, both domestically and internationally [19, 20].

For children with HCP, rather than representing ‘reparative plasticity’, enhanced ipsilateral projections from the intact cortex could worsen disability since they competitively displace the surviving contralateral cortico-spinal projections from the affected hemisphere [34]. Therefore, low-frequency rTMS was selected in this study to stimulate the contralateral cerebral hemisphere to inhibit the excitability of the contralateral cerebral cortex, weakening the inhibitory effect of the contralateral brain reduction on the affected side and enhancing the excitability of the affected cerebral cortex to promote the recovery of lower limb motor function on the affected side. Although the representative areas of the thumb and ankle in the brain motor cortex of children may overlap, and many studies have targeted the cortical area or bilateral cortical motor areas of the abductor pollicis brevis, researchers have observed an improvement in motor function after rTMS treatment [34, 35]. As the present study mainly observed the motor function of the lower limbs of children with HCP, the target was selected as the cortical area in the primary motor cortex that can stimulate the tibialis anterior to produce MEP.

After 4 weeks of rTMS combined with conventional physical therapy, the 10MWT, 6MWD, GMFM and the symmetry ratio of the step length and stance time of the two groups were significantly improved, with the results in the experimental group being significantly better than those in the control group. After rTMS combined with conventional physical therapy, the walking speed, walking endurance and GMFM of the participants improved significantly, and the step length and stance time of both lower limbs tended to be further symmetrical. In addition, this study observed a significant change in the tone of the triceps surae on the affected side, but no improvement in the hamstrings was observed.

Parvin et al. used rTMS combined with occupational therapy (OT) to treat a 13 years and two months with HCP and found improvements in 10MWT, Timed Up and Go test and 6MWT results. Furthermore, after evaluating the excitability of the reflex through the H-reflex response, it was found that excitability also improved [36]. This result is consistent with the research results of our study. We observed an improvement only in the muscle tone of the triceps surae and not the hamstrings. Although the lower limb muscles may overlap in the representative areas of the cortex, studies have shown that in most children under the age of 10, the phenomenon of proximal muscles being controlled by the uncrossed corticospinal tract is more common compared with distal muscles [37]. In our study, rTMS stimulated more of the cross-cortico-spinal tract, so it may have had a greater impact on the triceps surae at the far end. In addition, the sample size of this study was relatively small, and no changes in the muscle tone of the hamstrings were observed. Therefore, the results should be interpreted with caution.

Dadashi et al.’s study indicated that compared with using OT alone, rTMS treatment can improve both the therapeutic effect of OT treatment and the dynamic balance of children with CP [38]. Liang’s research also demonstrates that when low-frequency rTMS acts on the non-affected side of the cerebral cortex, it reduces its inhibitory effect on the affected side and promotes the function of the bilateral cerebral cortex to become balanced, thereby effectively improving the range of motion of the ankle joint and the motor function of the lower limb and shortening the recovery of motor balance function [39]. Although the balance function of the participants was not measured in this study, the D-zone (standing) and E-zone (walking, running and jumping) GMFM scores in the experimental group were significantly improved, and the muscle tone of the triceps surae and the symmetry ratio of the stance time were significantly enhanced. These results indicated that the control ability of the affected ankle joint and the lower limb weight-bearing ability of the experimental group were improved, as were the coordination and stability of both lower limbs [40]. This outcome can improve the walking efficiency and walking endurance of children with HCP, enable them to walk long distances and improve their independent living ability and quality of life.

Although our study confirmed significant immediate benefits of rTMS treatment in children with HCP, there were some limitations. First, the sample size was small, and, as such, the findings may have been subject to error. Second, there was no stratification of the differences in brain injury among individual participants, and the protocol is not yet perfect. Additionally, as the research indicators of GMFM and muscle tone are qualitative assessments of scales, there was subjective bias. More objective and direct assessment methods (such as surface electromyography, musculoskeletal ultrasound and functional magnetic resonance imaging) should be introduced in follow-up research. In future clinical studies, it will be necessary to continuously increase the sample size and complete follow-up to provide a basis for establishing the optimal mode of TMS treatment for children with HCP.

## Conclusion and clinical significance

This study found that rTMS combined with conventional physical therapy could improve the efficacy of neurological rehabilitation in children with hemiplegic cerebral palsy. This finding is of great significance for the clinical rehabilitation treatment of children with HCP and can be used as an auxiliary means of rehabilitation in the future. This study provides a reference and basis for the application of rTMS treatment in the rehabilitation treatment of children with HCP.

## Data Availability

All of the material is owned by the authors and/or no permissions are required.

## Abbreviations

rTMS: repetitive transcranial magnetic stimulation
HCP: hemiplegic cerebral palsy
10MWT: 10-meter walk test
6MWD: 6-minute walk distance
GMFM: gross motor function measure
MAS: modified Ashworth scale
CP: Cerebral palsy
CNS: central nervous system
GMFCS: Gross Motor Function Classification System
RMT: resting motor threshold
MEP: maximum motor evoked potential
OT: occupational therapy
Ltd: Limited Liability Company
